# Inhalation of Isopropyl Alcohol Leading to Acute Severe Toxicity: A Case Report

**DOI:** 10.1002/ccr3.72595

**Published:** 2026-04-24

**Authors:** Ben Thiravetyan, Chanokporn Puchongmart, Joscilin Mathew, Nattanicha Chaisrimaneepan, Cristian Rodriguez, Allan Bueso, Diego Rielo, Diego Cruz

**Affiliations:** ^1^ Department of Internal Medicine Texas Tech University Health Science Center Lubbock Texas USA; ^2^ Department of Internal Medicine, Division of Pulmonology Texas Tech University Health Science Center Lubbock Texas USA

**Keywords:** acute toxicity, hemodialysis, inhalation exposure, isopropyl alcohol

## Abstract

This case represents the first reported instance of adult isopropyl alcohol inhalation intoxication with a rapid onset (2 h) and initially negative serum and urine ketones. It highlights that early isopropyl alcohol intoxication may not exhibit the typical clinical features, such as respiratory depression, altered mental status, and positive ketones.

## 1 Introduction

Ingestion of toxic alcohols is known to cause acute toxicity characterized by a high osmolal gap [1]. The clinical presentation varies with each type of alcohol. Methanol ingestion can lead to severe metabolic acidosis and blindness, while propylene glycol can result in severe acidosis and the presence of calcium oxalate in the urine [1]. In contrast, isopropyl alcohol typically causes central nervous system depression without accompanying acidosis [1]. Isopropyl toxicity is known to occur through ingestion, transdermal absorption, and inhalation. Notably, isopropyl alcohol is one of the most commonly used household antiseptics in the United States, widely available as rubbing alcohol for disinfection and cleaning purposes [2]. Recent epidemiologic data from the 41st Annual Report of America's Poison Centers National Poison Data System (2023) demonstrate that household cleaning substances rank among the top contributors to serious poisoning outcomes, accounting for approximately 7.12% of major outcomes nationwide [3]. Despite its widespread availability and frequent use, there have been no well‐documented cases of severe intoxication due to inhalational exposure in adults [1, 4, 5].

Therefore, this case highlights a rare and clinically important presentation of isopropyl alcohol intoxication via inhalation, expanding the current understanding of its toxicokinetics and potential risks in real‐world settings.

## 2 Case History and Examination

A 69‐year‐old female with a medical history of hypertension and hypothyroidism presented to the emergency department with altered mental status for the past 2 h. Her family reported that she was scheduled for a colonoscopy for internal hemorrhoids the following morning and had taken polyethylene glycol as prescribed for bowel preparation earlier in the evening. After taking the medication, she napped and was found unresponsive 2 h later. Additionally, her sister reported that prior to taking the laxatives, the patient had used two 32‐oz bottles of rubbing alcohol to clean under the bedsheets to eliminate bed bugs and subsequently slept for a few hours in an enclosed room. Upon arrival at the emergency department, her vital signs were as follows: temperature 96.0°F, heart rate at 96 beats per minute, respiratory rate 28 per minute, blood pressure 110/73 mmHg, and SpO_2_ 97% on 4 L/min of oxygen nasal cannula. The patient was unresponsive, with an E2V2M4 score, pupils 3 mm, and reactive to light; no neck stiffness was noted, but arousable to pain stimuli, with equal extremity movements. However, motor power could not be assessed due to her uncooperativeness. Heart sounds were normal without a murmur, lungs were clear on auscultation, and the abdomen was soft and nontender. Laboratory evaluation revealed a complete blood count and serum chemistries that were unremarkable, including serum ketone. Serum glucose was 117 mg/dL. Urine toxin screen for amphetamines, barbiturates, cannabinoids, cocaine, and opioids was negative. Arterial blood gas showed pH 7.39, pCO_2_ 33.8 mmHg, pO_2_ 93.4 mmHg, HCO_3_ 20.4 mmol/L. Urinalysis was unremarkable, with negative urine ketone. Imaging studies included unremarkable computed tomography without contrast, computed tomographic angiography, and magnetic resonance brain imaging, all showing mild cerebral volume loss. EKG revealed normal sinus rhythm. EEG was suggestive of encephalopathy without an epileptic pattern. Salicylate levels were not detected. Echocardiography showed trace tricuspid and mitral regurgitation, trace pulmonic valvular regurgitation, and an ejection fraction greater than 70%. The patient was admitted to the intensive care unit due to severe altered mental status and was initially treated for a suspected systemic infection with empirical antibiotics.

## 3 Differential Diagnosis, Investigations, and Treatment

The patient initially presented with an acute onset of altered mental status, coupled with a significant history of bowel preparation using polyethylene glycol and cleaning under the bedsheet with rubbing alcohol. The differential diagnosis included intracranial pathologies such as acute stroke, seizure, or encephalopathy. After an extensive evaluation, no significant intracranial abnormalities were observed. Despite 20 h of antibiotic treatment, the patient showed no clinical improvement, and initial lab results were unremarkable. Subsequent serum osmolality testing revealed a marked osmolal gap of 65 mOsm/kg. Serum isopropyl alcohol was detected at 285 mg/dL, along with positive serum ketones, while methanol and ethyl alcohol were absent. The patient's family consented to hemodialysis. Thirty minutes after starting hemodialysis, the patient regained consciousness, became responsive, and was oriented to time, place, and person. Follow‐up lab tests showed a resolution of the elevated osmolal gap.

## 4 Conclusion and Results (Outcome and Follow‐Up)

The patient was finally diagnosed with acute isopropyl alcohol inhalation toxicity, which improved after a single session of hemodialysis. She was discharged the following day without complications and returned to the follow‐up clinic 2 weeks later, resuming all normal activities.

## 5 Discussion

Isopropyl alcohol is widely used as rubbing alcohol in the United States. It is commonly utilized as a cleaning agent in hospitals and is also available over the counter for general use. While isopropyl alcohol toxicity typically causes acute altered mental status, respiratory depression, and positive serum or urine ketones without a wide anion gap metabolic acidosis, it is generally associated with a low mortality rate [1, 4, 5]. In this case, a patient presented with acute isopropyl alcohol intoxication, with symptoms occurring within 2 h of exposure. After 20 h, the patient's serum isopropyl alcohol level was 285 mg/dL, which suggests a concentration likely higher at the time of initial presentation. Given its half‐life of 2.5–8 h, the exact initial concentration is difficult to determine [6]. A previous study from Alexander and coauthors found that lethal serum isopropyl alcohol concentrations have ranged from < 100 to 3200 mg/L, with a mean of 1240 mg/L [7]. Isopropyl alcohol intoxication typically results from unintentional ingestion and transdermal absorption of isopropyl alcohol in humans; most cases involve mild toxicity [5, 8]. Fatal isopropyl alcohol inhalation was once reported in a neonate who was accidentally exposed when the substance was placed in the humidifier of a ventilator [9]. This case presents the first severe toxicity associated with inhaled isopropyl alcohol, with notable clinical symptoms including altered mental status, a serum isopropyl alcohol level of 285 mg/dL, and negative initial serum and urine ketones.

Isopropyl alcohol is a volatile solvent with a vapor pressure of approximately 33 mmHg at 20°C–25°C and an evaporation rate of 1.5–2.3 relative to n‐butyl acetate, allowing it to readily form airborne vapor at room temperature [6]. Isopropyl alcohol also has a Henry's law constant of about 8 × 10^−6^ atm·m^3^/mol, indicating its tendency to partition into the gas phase and contribute to inhalational exposure, particularly in poorly ventilated environments [10]. Once inhaled, isopropyl alcohol may enter the systemic circulation rapidly because it is a small molecule (molecular weight 60 g/mol) and lipophilic and can diffuse efficiently across the alveolar–capillary membrane, which has a large surface area and high blood flow. This pulmonary uptake may lead to faster systemic exposure compared with transdermal absorption, which typically requires prolonged skin contact, and ingestion, which involves gastrointestinal absorption and first‐pass metabolism [6, 8]. In a controlled human exposure study, Nelson and colleagues exposed volunteers to isopropyl alcohol vapor concentrations of 200, 400, and 800 ppm for 3–5 min and observed mild to moderate irritation of the eyes, nose, and throat at the higher concentrations, demonstrating physiologic effects even after brief inhalational exposure [11].

These initial negative ketones may reflect an early stage of toxicity, as isopropyl alcohol has a reported half‐life of approximately 2.5–8 h, with acetone levels typically peaking several hours after exposure [6]. However, acetone may become detectable within approximately 30 min after ingestion, as described in the physiologic study by Lacouture and colleagues [12]. The kinetics of isopropyl alcohol exposure via inhalation, however, remain poorly characterized.

## 6 Limitations

A limitation of this case is the absence of an initial serum isopropyl alcohol level at presentation due to the nonspecific clinical presentation and initially negative serum acetone results. These factors limited our ability to determine the precise timing of exposure and to perform a detailed kinetic analysis of isopropyl alcohol metabolism in this patient.

## Author Contributions

**Ben Thiravetyan:** conceptualization, supervision. **Chanokporn Puchongmart:** writing – original draft. **Joscilin Mathew:** validation, writing – review and editing. **Nattanicha Chaisrimaneepan:** supervision. **Allan Bueso:** writing – review and editing. **Diego Rielo:** writing – review and editing. **Diego Cruz:** writing – review and editing. **Cristian Rodriguez:** writing – review and editing.

## Funding

The authors have nothing to report.

## Consent

Written informed consent was obtained from the patient for the publication of this report.

## Conflicts of Interest

The authors declare no conflicts of interest.

## Data Availability

The data that support the findings of this study are available from the corresponding author upon reasonable request.
